# When Is Improvement Not? Worsening Scores after Grant Proposal Revision

**DOI:** 10.1128/msphere.00155-23

**Published:** 2023-04-10

**Authors:** Michael J. Imperiale, Arturo Casadevall

**Affiliations:** a Department of Microbiology and Immunology, and Rogel Cancer Center, University of Michigan, Ann Arbor, Michigan, USA; b Department of Molecular Microbiology and Immunology, Johns Hopkins Bloomberg School of Public Health, Baltimore, Maryland, USA

## EDITORIAL

We’ve all experienced or heard the same story. A grant proposal is submitted for review by an NIH study section. The reviews come back with a score that is great but doesn’t make the pay line. A revised application is submitted that addresses the concerns that were raised about the original application. The second reviews come back … and the score is worse … or the application doesn’t even get discussed. What happened? How can this be?

The grant application process, which includes the ability to revise reviewed but unfunded proposals, ought to provide an opportunity to improve the quality of the proposed research since the criticisms often identify important conceptual or methodological problems with the experimental plan. When a reviewer provides constructive feedback in the comments, the applicant should be able to benefit from that wisdom. Indeed, applicants work hard to respond to the reviews, oftentimes doing more experiments to include additional preliminary data that address the concerns and further support the feasibility of what is being proposed. Hence, for most applications one can anticipate that the revision process improves the proposed work. However, such improvements are sometimes not reflected in the subsequent score. Although we can accept that for some applications the revisions introduce new experimental foibles that would worsen the score, we suspect that this occurs in a minority of revised proposals. The message that is sent when the next review says the “previous reviewers’ concerns have been adequately addressed” but the score gets worse is: “We provided some advice on how to improve your application, you followed it, and now the application is not as good as the first time through.” This is unreasonable. Does this mean the reviewers’ comments were off the mark in the first review? That would be a serious concern.

Those of us who have sat on study section have heard the instructions from the NIH: each review cycle is independent, which means that new reviewers may have new concerns or view the work to be less important. We understand that when an application gets new reviewers, these could identify flaws that were not apparent in the first review, but when this happens it should be clearly stated. Today, reviewers are asked to comment on the quality of the revision, but they are not privy to the final score during the voting process, which is averaged after the meeting and known only to the study section scientific review officer. In those rare cases where the review of a revised proposal identifies a problem that was missed on the first review, a justification for a worse score should be specifically noted in the critique. This would help an applicant who receives a worse score after revision know what happened and maintain the credibility of the system.

When a grant proposal is reviewed by study section, the scores and the comments represent the consensus of the group, not simply the thoughts of the assigned reviewers. We know this because every nonconflicted member of the study section has the opportunity to discuss the proposal, to suggest edits to the comments, and to assign a score. Indeed, members are allowed to “vote outside the range” of what is recommended by the reviewers provided that they disclose their intentions, meaning the reviewers’ comments are not the be all and end all. The summary statement and the score represent the group assessment, not the individual reviews. This is apparent when there is a discrepancy between the severity of the criticisms and the score. Therefore, when a revised application comes back and addresses the concerns, it is responding to the concerns of the study section writ large, since the group determined the overall impact score. If it is agreed at the next round that those concerns were adequately addressed, as is often the case, and no new problems are identified, we would argue that the study section should give the proposal a better score. Whether there were new reviewers or not, the message to the applicant comes from the group.

In what other human endeavor does improvement in response to constructive advice lead to worse outcomes? When an ice skater is not landing jumps well and their coach gives them instruction, they fix it and get better scores, even from different judges. If an English professor reads a student’s paper and makes suggestions to improve their writing skills, the student gets a better grade if they follow the advice, including in subsequent classes with different professors.

Most recently, we have heard that the phenomenon of worsening scores after revision is also occurring with F award applications from students. Imagine the message it sends to a graduate student or postdoc when they address the previous reviews and then are told, “You are now less qualified than the first time around.” Is that going to encourage young scientists to pursue a research career? No.

Over the years, many suggestions have been made on how to improve the peer review process, including by one of us, who suggested discarding the current scoring system for a modified lottery (1). These often require fairly substantive changes. There is a relatively simple solution to the worsening score issue, however: we all need to agree that if an applicant addresses concerns, the score should not get worse. In those rare cases where a worse score is clearly justified, that ought to be explicitly agreed upon in real time by the study section as a whole and the rationale for the new score must be provided to the applicant. This would improve the process and is the humane thing to do.
